# Fully Automated Assessment of Cardiac Chamber Volumes and Myocardial Mass on Non-Contrast Chest CT with a Deep Learning Model: Validation Against Cardiac MR

**DOI:** 10.3390/diagnostics14242884

**Published:** 2024-12-21

**Authors:** Ramona Schmitt, Christopher L. Schlett, Jonathan I. Sperl, Saikiran Rapaka, Athira J. Jacob, Manuel Hein, Muhammad Taha Hagar, Philipp Ruile, Dirk Westermann, Martin Soschynski, Fabian Bamberg, Christopher Schuppert

**Affiliations:** 1 Department of Cardiology and Angiology, University Heart Center Freiburg—Bad Krozingen, Medical Center—University of Freiburg, Faculty of Medicine, University of Freiburg, Südring 15, 79189 Bad Krozingen, Germany; 2 Department of Diagnostic and Interventional Radiology, Medical Center—University of Freiburg, Faculty of Medicine, University of Freiburg, Hugstetter Str. 55, 79106 Freiburg im Breisgau, Germany; 3 Siemens Healthineers, Siemensstr. 1, 91301 Forchheim, Germany; 4 Siemens Healthineers, 755 College Rd E, Princeton, NJ 08540, USA

**Keywords:** chest CT, non-contrast CT, cardiovascular diseases, incidental findings, deep learning

## Abstract

**Background**: To validate the automated quantification of cardiac chamber volumes and myocardial mass on non-contrast chest CT using cardiac MR (CMR) as a reference. **Methods**: We retrospectively included 53 consecutive patients who received non-contrast chest CT and CMR within three weeks. A deep learning model created cardiac segmentations on axial soft-tissue reconstructions from CT, covering all four cardiac chambers and the left ventricular myocardium. Segmentations on CMR cine short-axis and long-axis images served as a reference. Standard estimates of diagnostic accuracy were calculated for ventricular volumes at end-diastole and end-systole (LVEDV, LVESV, RVEDV, RVESV), left ventricular mass (LVM), and atrial volumes (LA, RA) at ventricular end-diastole. A qualitative assessment noted segmentation issues. **Results**: The deep learning model generated CT measurements for 52 of the 53 patients (98%). Based on CMR measurements, the average LVEDV was 166 ± 64 mL, RVEDV was 144 ± 51 mL, and LVM was 115 ± 39 g. The CT measurements correlated well with CMR measurements for LVEDV, LVESV, and LVM (ICC = 0.85, ICC = 0.84, and ICC = 0.91; all *p* < 0.001) and RVEDV and RVESV (ICC = 0.79 and ICC= 0.78; both *p* < 0.001), and moderately well with LA and RA (ICC = 0.74 and ICC = 0.61; both *p* < 0.001). Absolute agreements likewise favored LVEDV, LVM, and RVEDV. ECG-gating did not relevantly influence the results. The CT results correctly identified 7/15 LV and 1/1 RV as dilated (one and six false positives, respectively). Major qualitative issues were found in three cases (6%). **Conclusions**: Automated cardiac chamber volume and myocardial mass quantification on non-contrast chest CT produced viable measurements in this retrospective sample. **Relevance Statement**: An automated cardiac assessment on non-contrast chest CT provides quantitative morphological data on the heart, enabling a preliminary organ evaluation that aids in incidentally identifying at-risk patients who may benefit from a more targeted diagnostic workup.

## 1. Background

Non-contrast chest computed tomography (CT) has a well-established usage spectrum in clinical imaging. It is centered around the assessment of the airways and the lung parenchyma, complemented by an evaluation of the mediastinal lymph nodes to the extent they can be differentiated without intravenous contrast media. A cardiac assessment on non-contrast chest CT, however, is difficult to perform visually and is generally limited to gross organ enlargement, pericardial effusion, and signs of pulmonary congestion.

Automated cardiac segmentation algorithms can support readers by providing additional quantitative information and thus offer an opportunity for incidental cardiac evaluation. Depending on the accuracy of these data, it may help readers to identify cardiac abnormalities on non-contrast chest CT. Such an automated cardiac assessment could also be used as an opportunistic screening tool to identify asymptomatic patients potentially requiring further cardiac evaluation early. This may be especially beneficial for patients at risk for heart failure since they commonly develop symptoms only when cardiac function is already relevantly and irreversibly impaired. The prevalence of heart failure underlines the importance of this issue; in Europe, it is reported to be 1–2% in the general population [1,2,3], increasing to above 10% in the age group above 70 years [4,5].

Implementations of automated cardiac segmentation algorithms were long restricted in scope and precision: the assessment of single chambers, delineation along the epicardial contours, and a lack of myocardial mass quantification limited their diagnostic value [6,7,8]. A recently proposed method addresses these limitations by enabling segmentation of all four cardiac chambers along the endocardial border, complemented by a segmentation of the left-ventricular myocardium via delineation of the epicardial border [9]. In the technical report, segmentation results from non-contrast cardiac CT scans in the mid-diastolic phase were validated against those from paired and phase-matched contrast cardiac CT scans. Although the authors reported good agreement and correlation in this intramodal comparison, the method has not yet been validated through intermodal comparison with the clinical gold standard—cardiac MR (CMR)—for quantifying chamber volumes and myocardial mass, which is essential to confirm its clinical applicability.

Therefore, our objective was to assess the accuracy of an existing deep learning model for the fully automated quantification of cardiac chamber volumes and myocardial mass on non-contrast chest CT by comparing its results to clinical CMR as a reference standard.

## 2. Methods

### 2.1. Study Sample

**Figure 1:**
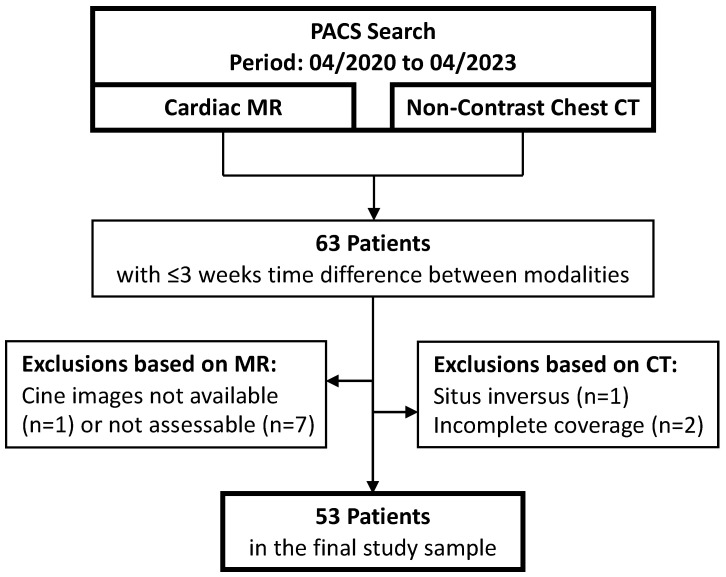
Flowchart of patient inclusion.

We queried the local picture archiving and communication system for all CMR, and non-contrast chest CTs performed as clinical examinations between April 2020 and April 2023. The results were matched at a patient level and pairs with an intermodal time difference of three weeks or less were kept. If a CMR or CT appeared in multiple examination pairs, the pair with the smallest intermodal time difference was selected to minimize temporal discrepancies. A general exclusion criterion was the presence of situs inversus. Exclusion criteria specific to CMR were non-assessment of the ventricles due to unavailability or incompleteness of short-axis cine sequences and severe image artifacts. Exclusion criteria specific to CT were examinations performed in other than the supine position and scan length not covering the full chest in the craniocaudal direction. The final study sample comprised 53 patients (Figure 1).

### 2.2. Patient Characteristics

The study sample comprised 19 female and 34 male patients. The mean patient age was 64 years. The mean BMI was 25.8 ± 5.3 kg/m^2^. The full patient characteristics are compiled in Table 1.

### 2.3. CMR: Imaging and Expert Segmentations as a Reference Standard

The CMR examinations included acquisitions on 1.5 T and 3 T scanners, specifically the 1.5 T models MAGNETOM Aera (*n* = 4), Avanto Fit (*n* = 2), and Espree (*n* = 10), as well as the 3 T models MAGNETOM Skyra (*n* = 13) and Vida Fit (*n* = 24) (all Siemens Healthineers, Erlangen, Germany).

The CMR image segmentations were performed in cvi42 (version 5.14, Circle Cardiovascular Imaging Inc., Calgary, AB, Canada). The LV epicardial, LV endocardial, and RV endocardial borders were automatically contoured in balanced steady-state free precession (bSSFP) technique short-axis cine sequences at end-diastole and end-systole. The left atrial (LA) and right atrial (RA) endocardial borders were automatically contoured in bSSFP long-axis cine sequences in four-chamber (both atria) and two-chamber (LA only) views at end-diastole to perform biplane or monoplane volume calculations, respectively. Two experts blinded to the diagnoses then assessed and, if necessary, manually corrected all segmentations in consensus: one board-certified radiologist with six years and one cardiology resident with three years of professional experience, including CMR. Severely interfering susceptibility artifacts warranted the exclusion of the affected chamber. The finally extracted parameters were LV and RV volumes at end-diastole and end-systole (MRLVEDV, MRLVESV, MRRVEDV, MRRVESV) for 53 patients, LA and RA volumes at ventricular end-diastole (MRLA, MRRA) for 52 and 49 patients, respectively, and LV mass at ventricular end-diastole (MRLVM, assuming a myocardial density of 1.05 g/mL) for 53 patients. The left and right ventricles were additionally classified as normal or dilated based on end-diastolic volumes normalized to the body surface area (BSA), following the CMR reference ranges of the European Association of Cardiovascular Imaging [10]: MRLVEDV ≤ 96 mL/m^2^ for females and ≤105 mL/m^2^ for males, MRRVEDV ≤ 112 mL/m^2^ for females and ≤121 mL/m^2^ for males. The left myocardium was classified as normal or hypertrophic with cutoff values of MRLVM ≤ 81 g/m^2^ for females and ≤85 g/m^2^ for males [10]. BSA was calculated using height and weight from the CMR examinations and the Mosteller formula [11].

### 2.4. Non-Contrast Chest CT: Imaging and Fully Automatic Cardiac Segmentations

The non-contrast chest CTs were acquired on two third-generation dual-source dual-energy scanners (SOMATOM Force, Siemens Healthineers, Erlangen, Germany). The scanning protocols included non-ECG-gated (*n* = 43) and prospectively ECG-gated (*n* = 10) variants. In the ECG-gated series, the targeted phase was 65% of the R-R interval and the measured phase ranged from 47% to 79%. The full scan parameters are listed alongside patient characteristics in Table 1. The mean difference in examination dates between CT and CMR was 3 ± 4 days (range 0–16 days).

For all CT examinations, a non-contrast axial image series with a soft-tissue reconstruction kernel and a thin slice thickness was processed using a deep learning segmentation model (AI-Rad Companion Research, Siemens Healthineers, Erlangen, Germany). The segmentation algorithm was trained on coronary CT angiography scans from a multi-center cohort of 2594 patients (mean age 60.2 ± 11.4 years, 45% female). Each scan comprised paired, cardiac-phase matched non-contrast, and contrast-enhanced series. Segmentation masks for the contrast-enhanced series were obtained using an automated approach and then registered to the non-contrast series through deformable registration to obtain ground truth segmentations for the cardiac chambers and the left ventricular myocardium. The quality of all ground truth segmentations on non-contrast scans was verified by human annotators before being used for model development. The deep learning model utilizes an image-to-image segmentation network, combined with a conditional variational autoencoder. Further details on the model architecture have been published by Jacob et al. [9]. In the present study, the model was used in combination with a heart isolation model to segment the cardiac chambers in non-contrast chest CT. Schematics of the training and inference pipelines are shown in Supplementary Figure S1. Algorithm development was done independently from and finalized prior to the results presented in this manuscript, and the training dataset did not include any data from the same institution as this validation study.

**Figure 2:**
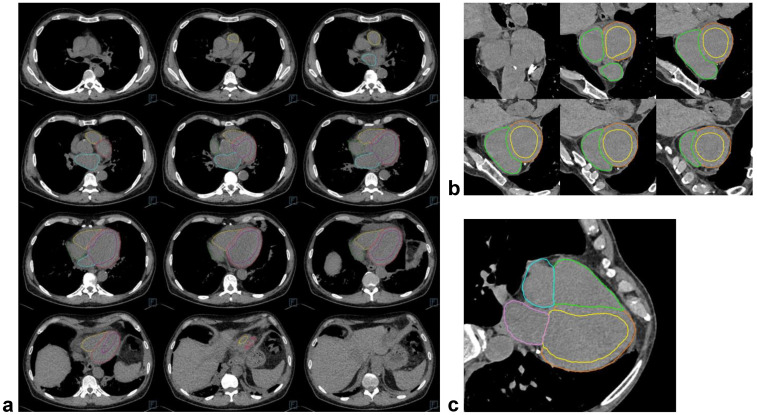
Examples of automatic cardiac chamber and myocardium segmentations on images from non-contrast chest CT scans. (**a**) Shows twelve axial slices from cranial to caudal for one example patient. (**b**,**c**) Show multiplanar reconstructions from another patient along the cardiac short-axis (ventricular base to apex, contours not displayed at the level of the annulus fibrosus due to the overlap of all four chambers) and long-axis in the 4-chamber view, respectively.

The algorithm output comprised the CT images (Figure 2) with a segmentation overlay and a set of derived parameters: LV (CTLV), RV (CTRV), LA (CTLA), and RA (CTRA) volumes, LV myocardial volume, and whole-heart volume. LV myocardial mass (CTLVM) was then calculated assuming the same myocardial density as for CMR, and all values were rounded to the nearest integer. All segmentations were visually assessed for plausibility and rated on a 3-point Likert scale: 1—no issues, 2—minor issues (limited to the cardiac structures and presumed not to induce a relevant volume error), 3—major issues (not limited to the cardiac structures or presumed to induce a relevant volume error). The effects of cardiac implants on the subjective segmentation accuracy were also noted. The density delta of the ventricular septum and left-ventricular blood pool was determined through two regions of interest at the basal to mid-ventricular level, sized as large as possible up to 100 mm^2^ and placed as accurately as possible given the non-contrast images. Additionally, the hematocrit from a blood sample close to the CT date (cutoff ≤ 5 days) was extracted from the clinical information system.

### 2.5. Statistics

Statistical analysis was conducted using SAS (version 9.4, SAS Institute Inc., Cary, NC, USA). Descriptive statistics were determined by means and standard deviations for continuous variables with normally distributed data or as percentages for categorical variables. Absolute differences between the CT-based assessment and the CMR reference were calculated as ‘CT minus CMR’. For the ventricles, the CMR reference included end-diastolic and end-systolic volumes, and the differences were calculated for both timepoints. For the subset of cases where CTLV or CTRV fell outside the respective reference range, the absolute differences to the nearest MR measurements were additionally calculated. Agreements between paired measurements were evaluated using Bland–Altman analytics and their differences were tested using the Wilcoxon signed-rank test or the paired *t*-test, depending on data distribution. Additionally, the within-subject coefficients of variance were calculated. The differences between independent measurements (esp. the influence of ECG-gated and non-ECG-gated acquisition techniques on measurement agreements) were tested using the Mann–Whitney U test or the unpaired *t*-test. Correlations between CMR and CT measurements were calculated using the intraclass correlation coefficient (ICC). ICC values were classified as “poor” (<0.50), “moderate” (0.50–0.74), “good” (0.75–0.90), or “excellent” (>0.90) [12]. Agreement between CMR and CT assessments for the categorical classification of ventricular volumes as normal or dilated was determined using percentages and binary confusion matrices in combination with the corresponding evaluation metrics. Univariable linear regression models were used to identify factors associated with the myocardial volume error between CMR and CT assessments. The level of statistical significance was set at *p* < 0.05.

## 3. Results

### 3.1. Availability of CT Measurements

The deep learning model generated CT measurements for 52 of the 53 patients (98%) included in the final study sample. These data, along with CMR measurements and selected statistical analysis results, are presented in Table 2.

### 3.2. Non-Contrast CT for the Assessment of Left Ventricular Volume and Myocardial Mass

**Figure 3:**
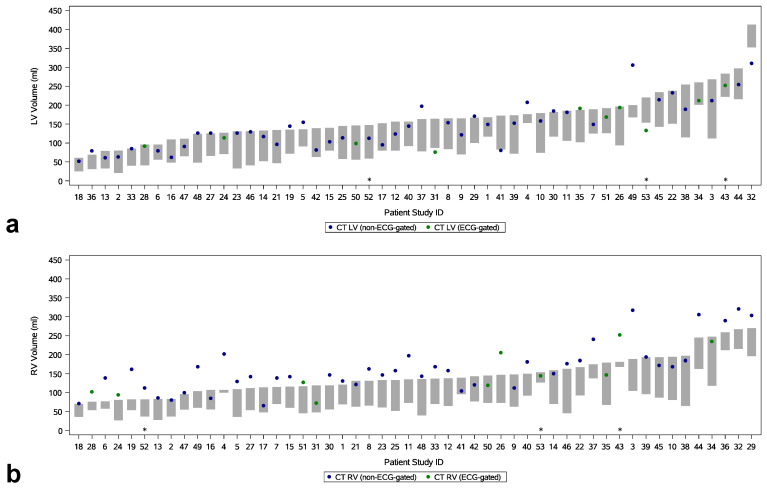
Range-based agreement for MR and CT segmentations. The MR segmentations (bars) included end-diastolic as well as end-systolic volumes for the left (**a**) and right (**b**) ventricles, thus providing a volume range within which the results from the automatic CT segmentation (dots) using non-phase-matched images were expected to lie. Asterisks: Cases with major CT segmentation failures in the visual quality assessment. LV: left ventricle, RV: right ventricle.

**Figure 4:**
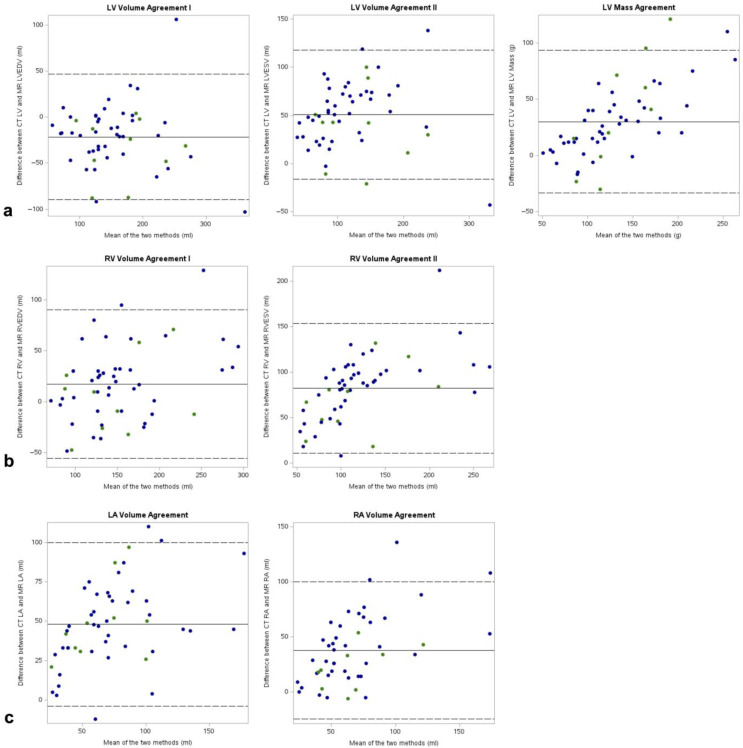
Bland–Altman plots for MR-based and CT-based measurements. (**a**) Left ventricular volumes and mass, (**b**) right ventricular volumes, (**c**) left atrial and right atrial volumes at ventricular end-diastole. LA: left atrium, LV: left ventricle, RA: right atrium, RV: right ventricle. Blue dots: Cardiac MR paired with non-ECG-gated chest CT. Green dots: Cardiac MR paired with ECG-gated chest CT.

**Figure 5:**
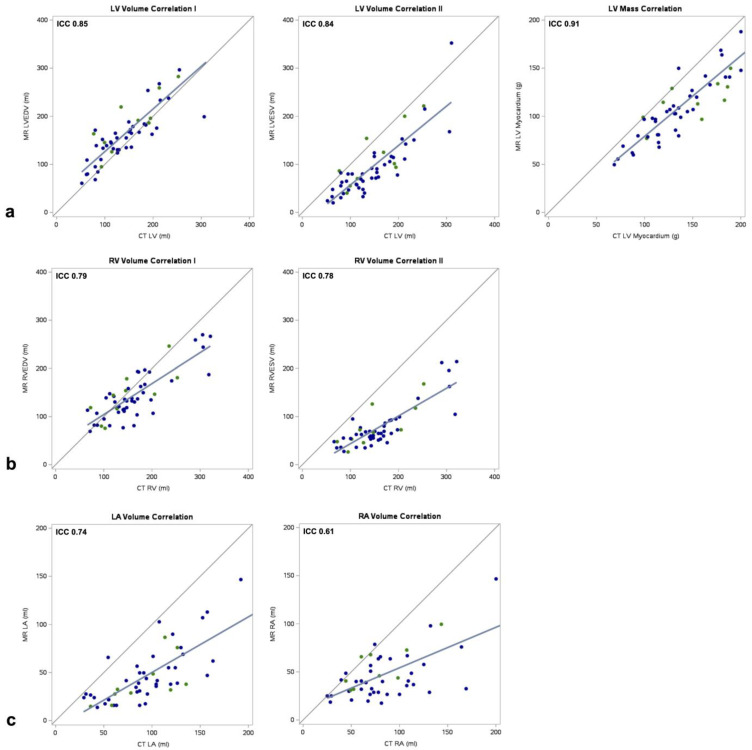
Scatter plots and correlation coefficients for MR-based and CT-based measurements. (**a**) left ventricular volumes and mass, (**b**) right ventricular volumes, (**c**) left atrial and right atrial volumes at ventricular end-diastole. LA: left atrium, LV: left ventricle, RA: right atrium, RV: right ventricle. Blue dots: Cardiac MR paired with non-ECG-gated chest CT. Green dots: Cardiac MR paired with ECG-gated chest CT.

In the overall sample, CTLV fell within the range of MRLVEDV and MRLVESV in 37 of 52 cases (71%) (Figure 3). The outlier measurements tended to overestimate, with a mean difference of 10 ± 33 mL (*p* = 0.22) between CT and CMR, equivalent to a mean relative volume error of +6.2%. There was no significant difference in adherence to the MRLVEDV-MRLVESV range between the ECG-gated and non-ECG-gated subsamples (ECG-gated: 70% of cases; non-ECG-gated: 71%, *p* = 0.15). The mean difference between CTLV and MRLVEDV was −22 mL (95% CI: −90 mL, 47 mL), and this tendency to underestimate did not differ with statistical significance between the subsamples (*p* = 0.31) (Figure 4). The agreement between CTLV and MRLVESV was inferior compared to MRLVEDV, with a mean difference of 51 mL (95% CI: −16 mL, 118 mL; *p* < 0.001). The correlations between CTLV with both MRLVEDV and MRLVESV were good (ICC = 0.85, *p* < 0.001 and ICC = 0.84, *p* < 0.001, respectively) and similar across subsamples for LVEDV (ECG-gated: ICC = 0.84, *p* = 0.003; non-ECG-gated: ICC = 0.85, *p* < 0.001) and LVSEV (ECG-gated: ICC = 0.79, *p* = 0.002; non-ECG-gated: ICC = 0.85, *p* < 0.001) (Figure 5). At the categorical level for classifying normal and dilated LV, the classification ability of the CT results was moderate: of the 15 patients with a dilated LV, seven (47%) were correctly identified, while eight were falsely classified as negative. One patient was falsely identified as positive (Supplementary Figure S2, Supplementary Table S1).

For the myocardial mass, CTLVM showed a tendency to overestimate, with a mean difference to MRLVM of 30 g (95% CI: −33 g, 93 g, *p* < 0.001). It did not improve with statistical significance in the ECG-gated compared to the non-ECG-gated subsample (*p* = 0.36) (Figure 4). The overall correlation coefficient was excellent at ICC = 0.91 (*p* < 0.001). The ECG-gated and non-ECG-gated subsamples showed disparate correlation coefficients (ICC = 0.62, *p* = 0.009 and ICC = 0.79, *p* < 0.001, respectively). At the categorical level for classifying normal and hypertrophic myocardium, the classification ability of the CT results was moderate: of the five patients with a hypertrophic LV, one (20%) was correctly identified, while four were falsely classified as negative. No patient was falsely identified as positive (Supplementary Table S1).

The individual LV components in terms of endocardial volume and myocardial volume showed inferior agreements and lower correlations than their combined total, represented by LV size, with a mean difference of 4.9 mL (95% CI: −74 mL, 84 mL) and a correlation coefficient of ICC = 0.92 (*p* < 0.001), using measurements at MR end-diastole. In univariate linear regression, none of the following predictors had an effect on the differentiation accuracy between LV cavity and myocardium: density difference (*p* = 0.87), hematocrit (*p* = 0.21), BMI (*p* = 0.45), tube current-time product (*p* = 0.66, volumetric computed tomography dose index (*p* = 0.44), ECG-gating (*p* = 0.85).

### 3.3. Non-Contrast CT for the Assessment of Right Ventricular Volume

In the overall sample, CTRV fell within the range of MRRVEDV and MRRVESV in 31% of cases (Figure 3). The outlier measurements all overestimated the RV volume, with a mean difference of 36 ± 29 mL between CT and CMR (*p* < 0.001), equivalent to a mean relative volume error of +27.5%. There was no significant difference in adherence to the MRRVEDV-MRRVESV range between the ECG-gated and non-ECG-gated subsamples (ECG-gated: 50%, non-ECG-gated: 26%, *p* = 0.98). The mean difference between CTRV and MRLVEDV was 18 mL (95% CI: −55 mL, 91 mL), and this tendency to overestimate did not differ with statistical significance between the subsamples (*p* = 0.21) (Figure 4). The agreement between CTRV and MRRVESV was significantly inferior compared to MRRVEDV, with a mean difference of 82 mL (95% CI: 11 mL, 154 mL; *p* < 0.001). The correlations between CTRV with both MRRVEDV and MRRVESV were good (ICC = 0.79, *p* < 0.001 and ICC = 0.78, *p* < 0.001, respectively) and similar across subsamples for RVEDV (ECG-gated: ICC = 0.77, *p* = 0.004; non-ECG-gated: ICC = 0.80, *p* < 0.001) and RVSEV (ECG-gated: ICC = 0.76, *p* < 0.001; non-ECG-gated: ICC = 0.79, *p* < 0.001) (Figure 5). At the categorical level for classifying normal and dilated RV, the single patient with a dilated ventricle was correctly identified, while six patients were falsely classified as positive (Supplementary Figure S2, Supplementary Table S1).

### 3.4. Non-Contrast CT for the Assessment of Atrial Volumes

In the overall sample, CTLA and CTRA were predominantly higher than MRLA and MRRA, with mean differences of 48 mL (95% CI: −4 mL, 100 mL, *p* < 0.001) for the LA and 38 mL (95% CI: −24 mL, 100 mL, *p* < 0.001) for the RA (Figure 4). The agreements between the ECG-gated and non-ECG-gated subsamples did not differ with statistical significance for either the LA (*p* = 0.83) or the RA (*p* = 0.14). The CTLA and CTRA measurements showed moderate correlations with MRLA and MRRA via coefficients of ICC = 0.74 (*p* < 0.001) and ICC = 0.61 (*p* < 0.001), respectively (Figure 5). For LA volumes, the correlation was stronger in the non-gated subsample (ECG-gated: ICC = 0.63, *p* = 0.003; non-ECG-gated: ICC = 0.75, *p* < 0.001), whereas for the RA volumes, it was stronger in the gated subsample (ECG-gated: ICC = 0.74, *p* = 0.005; non-ECG-gated: ICC = 0.61, *p* < 0.001).

### 3.5. Visual Quality Assessment of the CT Segmentations

The CT segmentations were without quality issues in 38 of 52 cases (73%). Minor issues were seen in 11 cases (21%) for one or two chambers in the form of small undersegmentations or oversegmentations into epicardial fat, pericardial effusion, and pericardial fat, or slight segmentation irregularities along the interventricular septum. Major issues were found in three cases (6%) and either involved the LV contours missing three caudal slices, the LV epicardial contour extending into an adjacent rib, or the RA contour extending significantly into the pericardial effusion. These cases included two ECG-gated and one non-ECG-gated examination. The two major oversegmentations of the LV affected cases with dilated LV cavities, one of which was incorrectly classified as normal by the CT-based assessment. The presence of cardiac implants in two cases, comprising an LV patch and leads from a cardiac resynchronization therapy device, did not appear to negatively affect the segmentation quality.

## 4. Discussion

We assessed the accuracy of a fully automated algorithm for the quantification of cardiac chamber volumes and myocardial mass in non-contrast chest CT. A quantitative and qualitative analysis against a CMR reference demonstrated solid performance for ventricular volumes and myocardial mass, albeit with statistically significant tendencies towards overestimation for the RV volume and myocardial mass. A moderate performance was observed for atrial volumes. A subgroup analysis stratified by ECG-gated and non-ECG-gated acquisitions did not reveal statistically significant differences between the groups.

Previously published studies demonstrated that the automated segmentation of cardiac chamber volumes and other substructures is feasible in contrast-enhanced images from cardiac CT or CT pulmonary angiography [13,14,15,16]. Other approaches capable of processing non-contrast CT images, some with the ability to discriminate the myocardium, were primarily designed for radiotherapy planning and have not been evaluated for accuracy in volume and mass quantification [8,17,18,19,20]. A recent method demonstrated excellent estimation of myocardial mass on non-contrast coronary calcium score CT scans [21]. The algorithm used in the present paper permits a more comprehensive cardiac assessment on non-contrast CT images that encompasses quantification of all cardiac chamber volumes and myocardial mass. A recent retrospective study validated its technical accuracy on 420 image series from non-contrast cardiac CT paired with contrast-enhanced coronary CT angiography as a phase-matched ground truth [9]. The observed correlation coefficients for ventricular and atrial volumes ranged between 0.92 and 0.98. The testing did not include image series from non-ECG-gated CT. However, a validation against CMR, which is the clinical gold standard for the assessment of cardiac function and structure, has not yet been performed.

Based on the results of our study, there is a good linear correlation with measurements from CMR. The observed trend towards overestimation of volumes and mass may be partially compensated for by multiplying the results with different constants. The potential to produce false positive results for dilatation may be favorable or unfavorable in a screening tool, depending on the preference for higher sensitivity or specificity. Notably, there was no significant volume error in the assessment of LV volumes. Moreover, no patient in this study received a false negative classification for LV dilatation. The performance deficit in assessing atrial volumes compared to ventricular volumes may partly stem from the one-dimensional monoplane and two-dimensional biplane volume estimation methods used in CMR, which can underestimate atrial volumes due to geometrical assumptions [22,23,24]. Consequently, it appears plausible that the three-dimensional CT-based assessment of atrial volumes in this study was more accurate than the CMR-based reference.

Using a different model than the present study, Naghavi et al. extracted cardiac chamber volumes and myocardial mass from non-contrast coronary artery calcium CT scans acquired in the MESA trial and found that this additional information significantly enhanced traditional calcium score-only risk prediction for cardiovascular events and all-cause mortality [25]. The authors also demonstrated that automatic left atrial volumetry from these non-contrast CT scans predicted incident atrial fibrillation and stroke over 15 years with performance comparable to manual left atrial volume measurements obtained from CMR [26]. The current study used non-contrast chest CT, which is frequently performed in clinical service and has a central role in the ongoing introduction of imaging-based lung cancer screening programs. These datasets are continuously acquired in large numbers; however, as is the case with non-contrast coronary artery calcium CT scans, information on cardiac chamber volumes and mass is most often lost since it is not visually accessible. The potential value of filling this information gap by using an automated quantification algorithm is underlined further by the high prevalence of subclinical cardiac dysfunction or heart failure in the general population, which poses a major health burden. The associated 5-year mortality rate is reported to be 67% [27]. A parallel screening for signs of heart failure, although not providing an ejection fraction, may, therefore, benefit a significant number of patients or screening participants. Miller et al. made progress in this area through the automatic segmentation of cardiac structures on low-dose, non-contrast, non-gated chest CT scans in the NLST trial [28]. The biomarker estimates were predictive of all-cause and cardiovascular mortality.

Future prospective studies could expand upon these endpoints by assessing the ability of these methods to opportunistically identify patients who may benefit from dedicated cardiac evaluation. Possible applications lie in both routine and emergency settings. Since cardiac dysfunction can be an adverse effect of cancer treatments like anthracycline chemotherapy, an automated cardiac assessment could also be useful in CT performed for oncologic staging. It would support heart failure screening via echocardiography and complementary tests (e.g., NT-proBNP) conducted before, during, and after the application of cancer treatments [29,30].

The following limitations of our study deserve consideration: The limited sample size of 53 retrospectively included patients restricts sample homogeneity and results in the underrepresentation of pathologies, which constitutes the primary limitation of the study. A larger sample size would have enabled more selective patient inclusion. As known from an earlier analysis, segmentation of cardiac substructures on non-contrast CT images is difficult primarily due to the lack of visibility of the endocardial border [7]. In our sample, the relatively constant volume relation between LV myocardium and LV blood pool meant the accuracy of the segmentation model when faced with a pathologically thickened myocardium was not assessed. Nonetheless, the training dataset did include a substantial share of patients with LV hypertrophy [9]. Depending on the population the sample is drawn from, even a larger study may need an oversampling of cases with the relevant cardiac pathologies. Given a larger sample size, it will additionally be feasible to correlate the assessment precision with different severities of dilatation or hypertrophy. Another limitation is that we did not phase-match the CMR and CT image series to increase the precision of measurement comparisons. Nevertheless, this phase-matching would only have been obtainable in the ECG-gated subgroup. While using phase-matched images may have improved the correlations in our study, they likely would have stayed below those observed in the aforementioned study by Jacob et al., which used contrast-enhanced coronary CT angiography as a reference, as the intramodal comparison with another three-dimensional dataset will reduce inaccuracies. Furthermore, although we tested the model on images acquired with a variety of different scanning parameters, these all originated from the same scanner model. Yet once more, the algorithm was trained on a more comprehensive dataset [9]. Lastly, the density measurements of the ventricular septum and left-ventricular blood pool may have suffered from partial volume effects by unwillingly including portions of the adjacent right-ventricular and left-ventricular blood pools or papillary muscles, given that these measurements could only be performed approximately on the non-contrast CT images.

In conclusion, the evaluated model for automated cardiac chamber volume and myocardial mass quantification on non-contrast chest CT produced viable measurements in relation to the clinical gold standard of CMR in this retrospective sample. A prospective cohort could fully confirm its suitability for opportunistic screening in trials or routine clinical service to identify patients potentially benefiting from dedicated cardiac evaluation.

## Figures and Tables

**Table 1:** Patient characteristics and selected CT scan parameters for the study sample.

Parameter	Value
Gender (female)	19 (36%)
Age (years)	64 ± 16
Height (cm)	171 ± 10
Weight (kg)	76 ± 19
BMI (kg/m^2^)	25.7 ± 5.3
BSA (m^2^)	1.9 ± 0.3
ECG gating (n)	10 (19%)
Pitch Factor	1.5 ± 1.1
Peak Tube Voltage (kV)	
80	1 (2%)
90	16 (30%)
100	23 (43%)
110	6 (11%)
120	3 (6%)
150	4 (8%)
Tube Current-Time Product (mAs)	143 ± 104
CTDI_vol_ (mGy)	3.1 ± 1.6
Slice thickness (mm)	
0.60	2 (4%)
0.75	13 (25%)
1.00	37 (70%)
3.00	1 (2%)
Kernel	
Bf36	9 (17%)
Bf39	1 (2%)
Bf40	38 (72%)
Br40	2 (4%)
Bv36	3 (6%)

The data are presented as counts with percentages or as mean ± standard deviation. BMI: body mass index, BSA: body surface area, CTDI_vol_: volumetric computed tomography dose index, ECG: electrocardiogram.

**Table 2:** Results from cardiac chamber volume and myocardial mass quantification on cardiac MR and non-contrast chest CT.

Parameter	n	CMR	CT	*p*	Bland–Altman		WCV (%)	ICC
					Difference	Limits of Agreement		
LV volume (mL)	52	166 ± 64 (ED) 94 ± 60 (ES)	144 ± 61	<0.001 <0.001	−22 ± 35 51 ± 34	−90; 47 −16; 118	18.9 40.4	0.85 0.84
LV mass (g)	52	115 ± 39 (ED)	142 ± 42	<0.001	30 ± 32	−33; 93	18.7	0.91
RV volume (mL)	52	144 ± 51 (ED) 79 ± 44 (ES)	161 ± 64	0.001 <0.001	18 ± 37 82 ± 36	−55; 91 11; 154	18.9 53.7	0.79 0.78
LA volume (mL)	51	48 ± 31 (ED)	96 ± 42	<0.001	48 ± 26	−4; 100	56.5	0.74
RA volume (mL)	48	48 ± 27 (ED)	86 ± 43	<0.001	38 ± 32	−24; 100	47.2	0.61

The data are presented as counts (n), mean ± standard deviation for CMR and CT measurements, *p* values, Bland–Altman analytics (including differences and lower/upper limits of agreement), within-subject coefficients of variation (WCV), and intraclass correlation coefficients (ICC). CMR: cardiac magnetic resonance imaging, CT: computed tomography, ED: end-diastole, ES: end-systole, LA: left atrium, LV: left ventricle, RA: right atrium, RV: right ventricle.

## Data Availability

The measurement data generated during this study are not publicly available, but are available from the corresponding author on reasonable request. The imaging data are not available due to ethical and legal considerations. The version of the algorithm employed in this work is currently available for research only.

## Supplementary Materials

The following supporting information can be downloaded at: https://www.mdpi.com/article/10.3390/diagnostics14242884/s1, Figure S1: Overview of training and inference pipelines for CT image segmentation; Figure S2: Linear regression analyses of left and right ventricular volumes derived from non-contrast chest CT and cardiac MR segmentations (normalized to body surface area) with 95% confidence limits; Table S1: Classifications of normal and dilated ventricles and normal and hypertrophic left ventricular myocardium based on non-contrast chest CT and cardiac MR, using reference ranges from the European Association of Cardiovascular Imaging.

## Abbreviations

BMI	body mass index
BSA	body surface area
CMR	cardiac magnetic resonance imaging
CT	computed tomography
CTLA	CT-based measurement: left-atrial volume
CTLV	CT-based measurement: left-ventricular volume
CTLVM	CT-based measurement: left-ventricular mass
CTRA	CT-based measurement: right-atrial volume
CTRV	CT-based measurement: right-ventricular volume
ECG	electrocardiogram
ICC	intraclass correlation coefficient
LA	left atrium
LV	left ventricle
MR	magnetic resonance (imaging)
MRLA	MR-based measurement: left-atrial volume
MRLVEDV	MR-based measurement: left-ventricular end-diastolic volume
MRLVESV	MR-based measurement: left-ventricular end-systolic volume
MRLVM	MR-based measurement: left-ventricular mass
MRRA	MR-based measurement: right-atrial volume
MRRVEDV	MR-based measurement: right-ventricular end-diastolic volume
MRRVESV	MR-based measurement: right-ventricular end-systolic volume
RA	right atrium
RV	right ventricle
