# Supplementary material for: Fully Automated Assessment of Cardiac Chamber Volumes and Myocardial Mass on Non-Contrast Chest CT with a Deep Learning Model: Validation Against Cardiac MR
Source: Diagnostics (Basel). 2024 Dec 21;14(24):2884. doi: 10.3390/diagnostics14242884 (PMC11675647; doi:10.3390/diagnostics14242884)
Supplement: Supplementary file 1 [file diagnostics-14-02884-s001.zip › diagnostics-3357539-supplementary.pdf]

Supplementary Material

Supplementary Figure S1. Overview of training and inference pipelines for CT image segmentation.

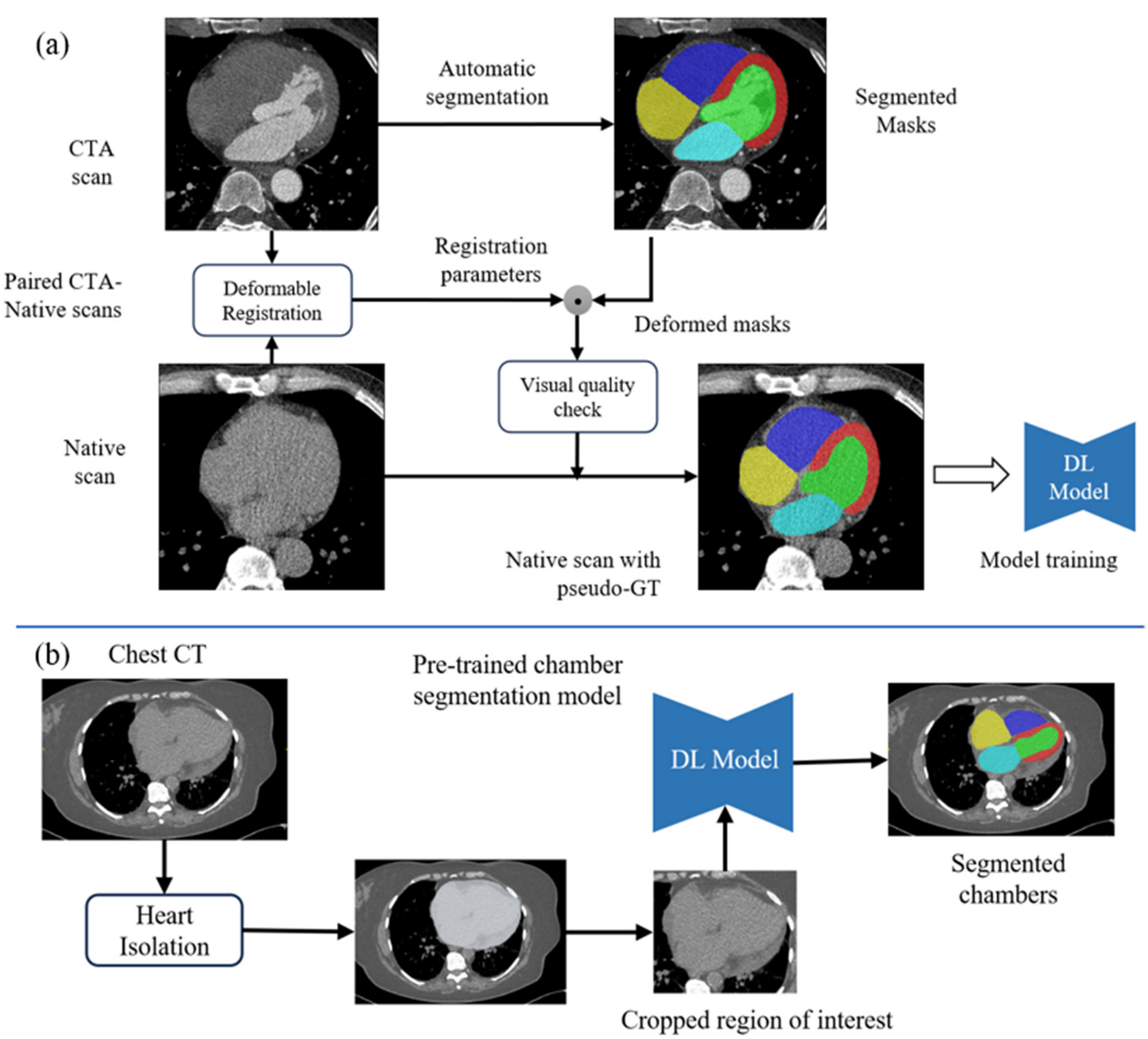

(a) A deep learning (DL) model was previously trained to segment cardiac chambers on non-contrast scans using paired coronary CT angiography (CTA) scans. (b) In this study, the model was used in combination with a heart isolation model to segment the cardiac chambers in non-contrast chest CT. GT: ground truth

Supplementary Figure S2. Linear regression analyses of left and right ventricular volumes derived from non-contrast chest CT and cardiac MR segmentations (normalized to body surface area) with 95% confidence limits.

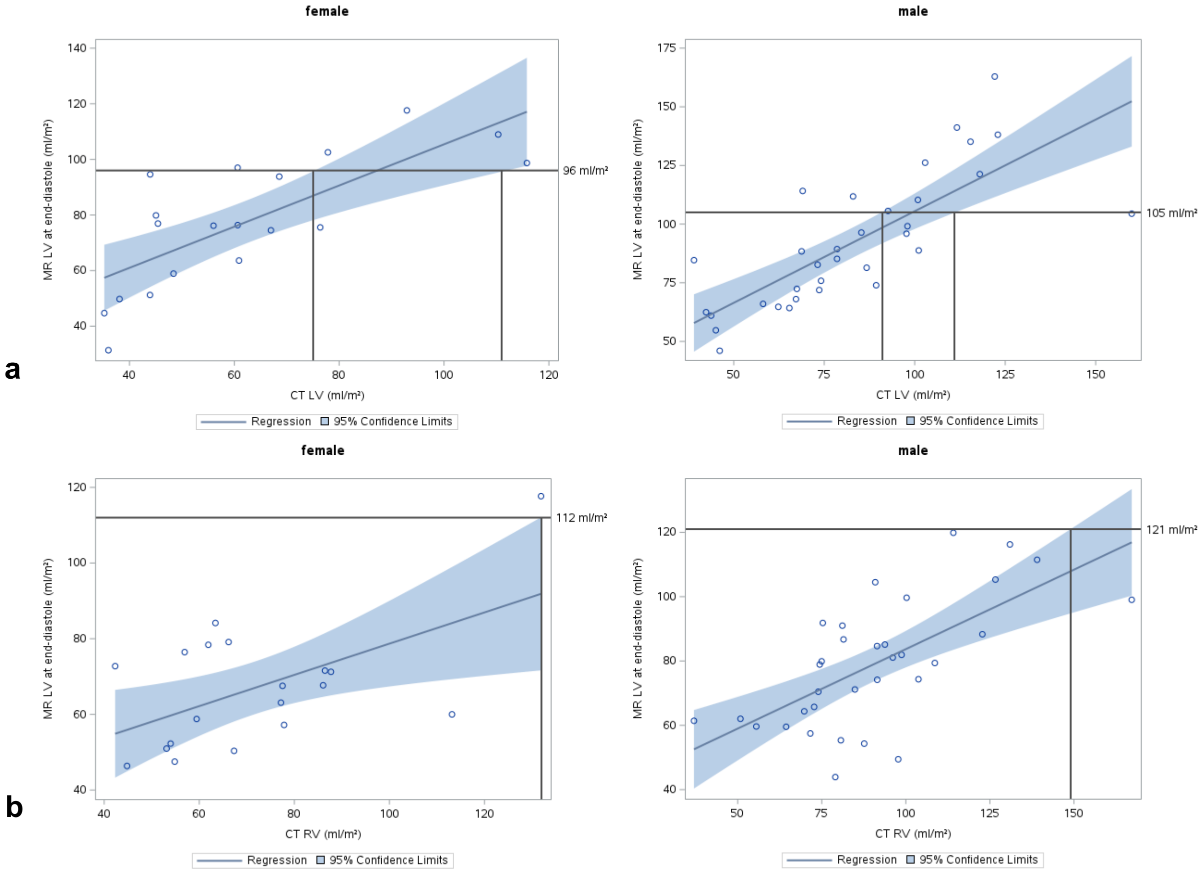

The black lines mark the upper reference range limits for end-diastolic volumes based on MR imaging according to the European Association of Cardiovascular Imaging. ***a*** *left ventricle (LV),* ***b*** *right ventricle (RV).*

Supplementary Table S1. Classifications of normal and dilated ventricles and normal and hypertrophic left ventricular myocardium based on non-contrast chest CT and cardiac MR, using reference ranges from the European Association of Cardiovascular Imaging.

**Left Ventricle: Volume**		**CT**		
		Dilated	Normal	
**CMR**	Dilated	7 (13 %)	8 (15 %)	Sensitivity: 0.467
	Normal	1 (2 %)	36 (69 %)	Specificity: 0.973
		Precision: 0.875	Negative Predictive Value: 0.818	Accuracy: 0.827

**Left Ventricle: Myocardium**		**CT**	
		Hypertrophic	Normal
**CMR**	Hypertrophic	1 (2 %)	4 (8 %)
	Normal	47 (90 %)	0

**Right Ventricle: Volume**		**CT**	
		Dilated	Normal
**CMR**	Dilated	1 (2 %)	0
	Normal	6 (12 %)	45 (87 %)

Left ventricular volume: The false positive case was from a non-ECG-gated examination, whereas the false negatives included both ECG-gated and non-ECG-gated examinations. Right ventricular volume: The false positive findings included ECG-gated and non-ECG-gated examinations. Measures of accuracy were not calculated for the left ventricular myocardium and the right ventricular volume due to the unsuitable numeric distributions. CMR: cardiac magnetic resonance imaging, CT: computed tomography
